# Respite time-use among dementia caregivers

**DOI:** 10.3389/frhs.2025.1598518

**Published:** 2025-07-28

**Authors:** Donald A. Godfrey, Bob Wong, Amber D. Thompson, Max E. Coleman, Catharine Sparks, Rebecca L. Utz

**Affiliations:** ^1^ College of Social and Behavioral Science, Psychology, University of Utah, Salt Lake City, UT, United States; ^2^ College of Nursing, University of Utah, Salt Lake City, UT, United States; ^3^ College of Social & Behavioral Science, Sociology, University of Utah, Salt Lake City, UT, United States; ^4^ College of Health, Occupational & Recreational Therapy, University of Utah, Salt Lake City, UT, United States

**Keywords:** caregiver stress, intervention, technology, family caregiving, time-use

## Abstract

Caregiving for family members with Alzheimer's disease and related dementias (ADRD) places significant burden on family members, leaving them at risk for a variety of mental and physical issues. While engaging in sufficient respite is generally considered an important resiliency factor for caregivers, recent research has demonstrated that caregivers are not satisfied with their respite and are not gaining much benefit during the limited respite time available to them.

**Objectives:**

The current study examines whether goal-oriented respite planning, facilitated by a mobile intervention, can improve caregivers' subjective experience of their respite time-use.

**Method:**

Caregivers (*N* *=* 85) used a mobile intervention to help them plan and evaluate their weekly respite time-use. Ecological Momentary assessments (weekly) monitored number of respite hours, respite goal achievement, and subjective assessment of their respite experience.

**Results:**

Respite goal achievement on a given week predicted improvements in participants’ ratings of their respite time-use outcomes one week later. Specifically, one week after reporting improved respite goal achievement, caregivers' ratings on happiness with their respite activities, feeling that their respite made them a better caregiver, and feeling like they had enough respite all increased. These effects were independent of the number of respite hours they reported per week.

**Discussion:**

Engaging in weekly goal-setting and goal-review activities is associated with caregivers’ subjective evaluation of their respite time-use. Interventions that help caregivers implement goal setting and achievement into their daily lives would likely benefit subjective evaluations and experiences with respite.

## 1 Introduction

Approximately one in five American adults care and support for a family member or friend with chronic illness or disability. For women and adults between the ages of 45–65 years old, prevalence of caregiving is even higher at nearly 25% (1). Caregiving efforts are a vital contribution to the nation valued at nearly $600 billion (2). These mostly unpaid caregivers provide an average of 24 h of care per week, extending over an average of 4.5 years per caregiving episode (3). They provide help with medical and nursing tasks; assistance with instrumental activities of daily living and household tasks such as cooking, transportation, and meals; decision making surrogacy; coordination and management medical appointments and care plans; as well as financial support, companionship, and advocacy for the person they are caring for (4).

Although many caregivers find significant meaning and value in the role (5, 6), it is not uncommon for caregivers to report feeling unprepared (7), or to experience considerable stress and burden, including financial hardships, placing them at risk of poor socio-emotional and physical and mental health outcomes (3, 8, 9), especially as the caregiving role extends over time (10). Those caring for persons living with Alzheimer's disease and related dementias (ADRD) report exceptionally high levels of daily stress (11), given the challenging symptoms and extended nature of the ADRD illness (12). Establishing ways to support and prepare family caregivers, especially ADRD caregivers, recognizes the undeniable public health value of family caregiving, while acknowledging caregivers' challenges of being unprepared, unsupported, and feeling over-burdened by the caregiving role (13).

Respite, defined as a break from caregiving responsibilities, is identified as one of the most needed, desired, and potentially beneficial services for caregivers (14, 15). Respite can be achieved through formal service, provided by adult day centers, in-home respite providers, or institutional and overnight settings. Respite may also be provided informally through shared arrangements that families, friends, and neighbors set up to ensure that the care recipient's needs are taken care of while the primary caregiver gets an occasional break (16). When scheduled regularly and in sufficient doses (17), respite provides caregivers a temporary break to focus on their own health, interests, or needs (18), an imperative if caregivers are going to be able to sustain the stresses of the caregiving role.

Research findings have produced inconsistent and mixed results on the overall benefits of respite to caregivers (19–21). For example, some caregivers did not feel comfortable having someone else look after their care recipient, felt guilty taking a break, or experienced anxiety that the care-recipient would have embarrassing or challenging behaviors while the caregiver was away (22, 23). Furthermore, almost half of caregivers reported dissatisfaction with how they spent their respite time, lamenting that they wasted time doing nothing, instead of using respite as a reprieve from their role as vigilant caregivers to pursue activities that are personally meaningful or rewarding (24). Those who used respite to do what they had most desired, needed, or had planned to do had the highest satisfaction with respite and also reported the most positive wellbeing over time (i.e., lower levels of burden and depressive symptoms) (15, 25). This finding emerged regardless of how much respite time one had, or what kind of activity the caregiver chose to do during respite – e.g., an obligatory chore that could be done during a break of only 15 min or a weekend-long recreational activity with a friend could each be seen as meaningful and beneficial, as long as it was what the caregiver chose and wanted to do with their time away.

These findings are consistent with a long-established principle from time-use research, which argues that congruence between desired and actual time-use is a significant predictor of overall life satisfaction (26). The American Time Use Study (ATUS) describes two dimensions of how people perceive their time-use: *Experienced time-use* measures “momentary positive and rewarding or negative and distressing states”, while *Evaluated time-use* refers to how specific time-use activities might shape one's “judgments of their overall life satisfaction or dissatisfaction” (27). Applied to respite research, these constructs underscore the importance of understanding what caregivers report doing during their respite time (i.e., “waste their time”), and also how caregivers both *experience* and *evaluate* their respite time-use. Accordingly, there has been a call for new research that explores respite time-use and respite planning/goal-setting as a determinant of respite satisfaction and benefit (28), signaling a move away from traditional methodological comparisons of respite users to non-users and research designs that focus on how much respite one had as the key predictor of overall benefit (29, 30).

Time for Living and Caring (TLC) is a caregiver intervention that focuses on respite time-use and respite planning, with the goal of maximizing caregivers' satisfaction and perceived benefit of respite, regardless of how much respite time they may have (25). Recently, the TLC intervention was adapted for self-administered delivery using an online mobile application [app; (31)], in response to a call for the use of technology to deliver support to caregivers (32).

TLC uses repeated goal-setting and goal-review exercises to help caregivers become more aware and planful about their respite time. A meta-analysis of 94 studies revealed that having a clearly stated goal and a realistic plan of action were positively related to successful goal attainment (33). Other past studies find evidence that engaging in the repeated process of goal-setting and goal-review can facilitate behavior change even when the person may be reluctant to change (33) – as is often the case among caregivers who are somewhat hesitant to even use respite – because the process of repeatedly specifying and revising goals, developing realistic and individualized implementation plans, and reviewing progress toward those goals increases self-awareness and reduces perceived barriers or hesitations (34, 35).

The objective of the current study was to conduct a pilot of the TLC app, specifically examining how respite planning and goal achievement via the app may affect caregivers' *experienced* and *evaluated* time-use. It is an exploration of how an “app” may be able to facilitate and train caregivers to become more proficient in these processes over time, thus leading to better respite time-use. The following hypothesis guides the current analysis: *Greater goal achievement will predict more positive respite time-use outcomes*. Thus, assuming that the TLC intervention can successfully coach caregivers to schedule and plan their respite in advance, they will be more likely to do the type of activities they planned to do, leading them to both experience and evaluate their respite time-use more favorably. Understanding this dynamic theoretically and empirically observing it within the context of the TLC pilot study is particularly important, as the TLC intervention is potentially a simple and novel intervention to empower caregivers to maximize the benefit of respite services, which are in high demand among caregivers who need and deserve a break.

## 2 Method

Data for the current study came from the TLC study, a pilot-test of the TLC mobile intervention with a sample of dementia caregivers.

### 2.1 Intervention

The TLC app consists of an initial assessment, an interactive calendar, and a series of 16 weekly coaching tips (i.e., automated and pop-up prompts) that facilitate goal-setting and goal-review activities that help caregivers schedule, identify, plan, and potentially modify how they use the respite time available to them. The intervention was designed on a weekly schedule, rather than the 24-hour diary approach commonly employed in time-use studies. This reduces burden associated with collecting traditional time-use data, a necessary modification for a highly time-burdened population like caregivers. Consistent with other psychosocial interventions, caregivers are expected to become more self-aware and independent in their respite planning over time (36).

First, an *initial assessment* helps caregivers identify specific activities once enjoyed but potentially sacrificed due to caregiving responsibilities and to brainstorm “wish list” activities they would like to do during respite but have not had time to accomplish because of their caregiving responsibilities. Next, the *Goal-Setting* prompts guide caregivers to schedule upcoming respite periods on the interactive calendar, and then to use a “SMART goals” framework (defined as goals that are specific, measurable, attainable, relevant, timely) (37, 38) to set time-use goals for each scheduled respite period. Additionally, weekly *Goal Review* module is used to ascertain whether the caregiver did the activity they had planned to do during their scheduled respite time (i.e., goal attainment), and also collects information on caregivers’ experienced and evaluated time-use assessments. These reflections (data) are visually displayed on a dashboard, helping caregivers become more self-aware of how the goal-setting and goal-review process is improving their respite time-use satisfaction over time. The TLC intervention provides caregivers an opportunity to engage in and practice the goal-setting and goal-review process for up to 16 weeks. More detail about the intervention can be found elsewhere (31).

### 2.2 Study design

Procedures for the larger TLC study were approved by the University of Utah Institutional Review Board. The TLC study used a longitudinal study design, where participants used the app for 16 weeks and completed surveys every 4 weeks. Consent for screening and participation was first obtained during initial phone calls, in which a research study team member would read the informed consent to interested individuals and consent was verbally confirmed during the initial phone call. Then, participants were sent a link to download a copy of the informed consent document, a link to the baseline questionnaire, as well as a link to access the TLC app. Participants could access the TLC app for 16 weeks, and completed self-report questionnaires every 8 weeks. Further details about the recruitment strategies and longitudinal design used for the larger TLC study can be found elsewhere (39).

The specific data used for this analysis includes self-report weekly assessments collected from participants as they used and engaged in the TLC app. We refer to these data as a form of Ecological Momentary Assessments (EMA), given that these data had the potential to be collected frequently and regularly. The EMA data produced by user-engagement of the TLC app is available up to weekly (e.g., thus up to 16 weekly EMAs per person). These data focus on how much respite time participants had, as well as their subjective evaluation and experience of their respite time-use during the previous week.

### 2.3 Sample

The TLC sample included primary caregivers to someone with Alzheimer's disease and related dementias who lived in the same house as the care recipient. For study inclusion, caregivers were required to have interest and ability to engage in respite at least once a week for a minimum of 4 h, speak English (because the mobile app was only created in English for this pilot test), and be over the age of 18. Potential participants were identified through a clinical database, word-of-mouth referrals, and other community-engaged recruitment methodologies (40). As shown in Table 1, the analytic sample used here (*N* = 85) is similar in demographic characteristics to the full TLC sample (*N* = 163), and is also in line with national populations of dementia caregivers. Means and proportions of demographic variables did not significantly differ between the full sample and EMA sample (*p*s > .05).

**Table 1:** Demographic and sample characteristics.

Caregiver characteristics	Current analytic sample	Full TLC sample	National sample of caregivers^a^
	*N* = 85 avg. (SD) or %	*N* = 163 avg. (SD) or %	11 million Americans %
Age (in years)	62.93 (13.92)	61.7 (13.0)	30% are over the age of 65
Sex – Female	76.5%	78.5%	61.5%
Race-White	91.3%	86.7%	66%
Ethnicity – Hispanic	3.7%	5.7%	8%
Education – college degree+	55.4%	55.6%	40% have college degree
Employment – currently employed	35.5	37.5%	Approximately 60%
Income adequacy – adequate or more	71.4%	81.4%	41% w incomes less than 50k^b^
Marital status – married or partnered	86.4%	83.8%	60%
Caring for spouse or partner	69.5%	70.6%	

There were no statistically significant differences in means or proportions across the current analytical sample and the full TLC sample.

^a^ Comparative statistics, when available, came from the 2023 Alzheimer's Association Annual Facts and Figures report (Alzheimer's Association, 2023).

^b^ There was not a comparable statistic for income: TLC study measured perceived income adequacy (is your income adequate to meet your needs), whereas the Alzheimer's Association data reported income.

Participants provided up to 16 Ecological Momentary Assessments (EMA) during the 16-week study period. Approximately 52% (*N* = 85 of 163) of the original TLC sample completed at least two EMAs over the course of the intervention period, a selection criterion for the analytic sample for this study. Excluded from the analytic subsample are those participants from the original TLC sample that had little to no engagement in this optional feature of the TLC intervention, which focused on recording specific respite time-use goals, planning and scheduling those goals, and reviewing whether they completed them the following week (i.e., they did not have any relevant repeated measures EMA data or enough data to be used for this analysis, *n* = 78 of 163). This suggests that this particular feature of the TLC app may not have been desired, preferred, or feasible for about half of the sample of caregivers in the larger TLC sample, raising future research questions about which types of caregivers may engage with which types of features on an app-delivered intervention (this is not a focus of this analysis). On average, caregivers included in this analytic sample completed 7.5 weekly reviews in the app (7.5 EMAs per person) across the 16-week observation and data collection period (range = 2–16). This means that the average caregivers who utilized the weekly review feature of the TLC intervention completed an EMA approximately every two weeks during the 16-week period. This biweekly pattern of engagement is what we benchmarked and expected prior to developing the study, and provides some empirical evidence for future studies about the level and frequency of engagement that might be expected for this type of population and this type of self-administered, technology-delivered intervention that allows for flexibility and personalization.

### 2.4 Measures

The outcomes for this study are related to caregivers' subjective experience and evaluation of *Respite Time-Use*; these measures were obtained from three self-report survey questions collected via the weekly EMA including: (1) “I am getting enough respite time”, (2) “I am happy with what I chose to do during my respite time”, and (3) “I am a better caregiver because of the respite I had this week”. Participants responded to each prompt on a Likert scale ranging from strongly agree (5) to strongly disagree (1). These measures were created specifically for the larger TLC study; they are intended to be analyzed separately for assessment of weekly ecological analysis. The first two questions (getting enough respite and happy with what I did during respite) are indicators of one's satisfaction with respite time-use or the *experienced* dimension of time-use, while the third question (i.e., better caregiver because of respite time-use) captures one's assessment of how their respite time-use made them feel or the *evaluated* dimension of time-use (27).

As part of the weekly review, participants engaged in a brief goal-review activity where they assessed, for each scheduled respite appointment from the week prior, whether they did the types of activities they had planned to do during that scheduled respite period. These data are used as the key independent variable that measures one's level of *Respite Goal Achievement*. The weekly review asked, “How successful were you in doing what you said you were going to do during [date/time – i.e., a scheduled respite period on the calendar]?” Responses ranged from 1 to 5 with higher scores indicating more success for achieving the specific respite time-use goal or planned activity: (1) not at all successful to (5) completely successful.

During the weekly EMA, participants confirmed how much respite they received during the prior week, in hours per week. This measure was self-reported by participants, who had access to an interactive calendar within the TLC app to facilitate their recall and scheduling of respite time each week. *Respite Time* is used as a time-varying independent variable or covariate in the current analyses.

A final variable – *Study Group* – was controlled in all analyses to account for features of the TLC study design. The TLC study employed a modified waitlist control design, where participants were randomized to treatment arms where they received the TLC intervention in different dosages over time. Both groups received access to all of the TLC coaching features and resources across the 16-week study period: the TLC-Immediate group received full access at week 1 that continued throughout the 16 weeks, while the TLC-Delayed group received a staggered delivery approach where certain features were provided at week 1 and others were unlocked at approximately 8 weeks. In this analysis, there were no statistically significant group differences in the processes and relationships explored; thus, analyses are reported for the full sample, and no group interactions were explored or presented.

### 2.5 Analysis

All questionnaire and study tracking data were stored in Research Electronic Data Capture (REDCap; UL1TR002538 NCATS/NIH). All data were cleaned and analyzed using Stata 16 (Stata, 2019) and SPSS, version 28.0 (IBM Corp.). Missing Values Analyses were conducted to explore if there were systematic patterns of missing data in the study overall. Missing values were found to be “Missing Completely at Random” for the “I am getting enough respite time” [χ^2^ (528) = 542.30, *p* *=* .32], “I am happy with what I chose to do during my respite time” [χ^2^ (528) = 567.29, *p* = .12], and the “I am a better caregiver because of the respite I had this week” prompts [χ^2^ (528) = 546.09, *p* = .28; (41)]. Therefore, no adjustments were made to models to correct for missing data.

Prior to hypothesis testing, study variables were inspected and descriptive statistics, bivariate correlations, and intraclass correlations were computed to characterize the data. We also used a growth model to estimate general trajectories in respite goal achievement to determine if individuals' ability to achieve their respite goals changed on average throughout the study period using multilevel growth modeling. To test our primary hypothesis, we used multi-level modeling approaches for intensive longitudinal data (42). Equation 1 provides a description of the model used to test primary hypothesis with *I* indexing week and *j* indexing participants. A random intercept (*β*_0*j*_) was used to separate variance in the outcomes between each participants' average scores (*μ*_2*j*_) across the weekly data collection intervals and differences within participants' own scores (*ɛ_ij_*). We disaggregated independent variables into two levels to distinguish within- and between-person fixed effects. At the within-person (level 1), fixed effects were person mean centered for both weekly goal achievement (*β*_2*j*_) and respite time (*β*_4*j*_). Positive values for these level 1 fixed effects can be interpreted as a weekly increase in goal achievement score or in hours of respite achieved per week, relative to a participant's average. Lagged effects of both variables were also included to determine if changes in goal achievement or respite time at a given week predicted experienced and evaluative respite time-use outcomes the next week (*t* *−* 1; *β*_3*j*_, *β*_5*j*_). To control for the influence of time and autoregressive effects of correlated variance in variables between adjacent time points, we included a lagged dependent variable that is also person mean centered (*β*_1*j*_), a growth parameter which indicates consistent weekly changes in outcomes (*β*_6*j*_), and an autoregressive residuals matrix (*r_ij_*).

At level 2, variables estimated differences between participants' average values in the study outcome variables. Fixed effects of goal achievement (*γ*_01_) and respite time (*γ*_02_) are calculated by computing the average of participant *j's* scores. These variables were then grand mean centered. Additionally, we included a dummy coded variable to control for differences in average scores based on whether individuals were in the delayed access or immediate access group. Lastly, goal achievement and lagged goal achievement fixed effects were allowed to vary between individuals with random slopes at level two (*μ*_2*j*_, *μ*_2*j*_).

Level 1:

$$\begin{array}{rl} \mathrm{Outcom}e_{ij} & =\beta_{0j}+\beta_{1j}*\mathrm{Dependent}\;\mathrm{variable}\;(t-1)\\ & +\beta_{2j}*\mathrm{Goal}\;\mathrm{Achievement}\;(t)+\beta_{3j}*\mathrm{Goal}\;\mathrm{Achivement}\;(t-1)\\ & +\beta_{4j}*\mathrm{Respite}\;\mathrm{Time}\;(t)\\ & +\beta_{5j}*\mathrm{Respite}\;\mathrm{Time}\;(t-1)\\ & +\beta_{6j}*\mathrm{week}+r_{ij}+\varepsilon_{ij} \end{array}$$

Level 2:

$$\begin{array}{rl} \beta_{0j} & =\gamma_{00}+\gamma_{01}*\mathrm{Average}\;\mathrm{Goal}\;\mathrm{Achievement}\\ & +\gamma_{02}*\mathrm{Average}\;\mathrm{Respite}\;\mathrm{Time}+\gamma_{03}*\mathrm{randomization}\;\mathrm{group}\\ & +\mu_{0j}\beta_{2j}\\ & =\gamma_{20}+\mu_{2j}\beta_{3j}=\gamma_{30}+\mu_{3j} \end{array}$$

### 2.6 Sample size consideration

Power to estimate significant effects was determined using recommendations from Arend & Schäfer (40). Given that we have 85 individuals with an average of 7.5 observations each and assuming moderate to large variability in random intercepts, we were able to detect, at minimum, a small (*β* *=* *.*16) effect for level 1 within person effects at 80% power. Additionally, we were able to detect, at minimum, a moderate effect (*β* *=* *.*35) for level 2 between person effects at 80% power.

## 3 Results

### 3.1 Descriptive statistics

Descriptive statistics, bivariate correlations, and intraclass correlations of the study variables are provided in Table 2. Participants had an average of 18.8 h of respite per week, and varied approximately ±8 h across the 16-week observation period. The intraclass correlations of respite time provided in Table 2 (ICC = .67) indicate that approximately 67% of the variability in scores was *between* individuals indicating that participants were moderately consistent in their weekly respite time hours. The average score from the experience and evaluative respite time-use outcomes fell between neutral (3) and agree (4).

**Table 2:** Descriptive statistics and correlations of study variables.

Study variables	Mean	*SD* between	*SD* within	1	2	3	4	5
1. Weekly respite time (in hours)	18.813	14.784	7.981	(.674)	.225***	.164***	.165***	.308***
2. Respite goal achievement (1–5)	3.933	0.541	0.677	.095*	(.316)	.803***	.762***	.636***
3. Happy with respite (1–5)	3.722	0.636	0.779	.153**	.456***	(.360)	.831***	.722***
4. Better caregiver because of respite (1–5)	3.766	0.608	0.655	.121**	.424***	.627***	(.420)	.582***
5. Enough respite (1–5)	3.377	0.735	0.865	.235***	.389***	.523***	.538***	(.370)

Upper diagonal – person average correlations; lower diagonal – within person changes correlations; middle diagonal – intraclass correlations.

* *p* < .05.

** *p* < .01.

*** *p* < .01.

**Figure 1:**
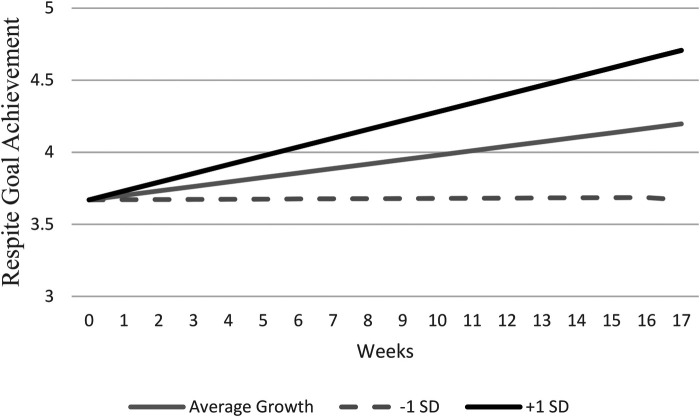
Growth model of goal achievement across study period.

When evaluating trajectories in goal achievement across the course of the data collection, we found that caregivers reported significant weekly increases in goal achievement (*b* *=* .03, *SE* *=* .01, *p* < .01). We included a random slope for the fixed effect of weekly changes, as there was significant variation in the linear increases among caregivers [μ = .004, *SE* *=* .002, 95% CI (−0.002, 009)] as those one standard deviation above average in growth experience reported an increase of.093 each week and those with one standard deviation below average in weekly growth reported experiencing a change of −0.030 each week. Additionally, there was significant covariability between the random intercept and slope [μ = −0.04, *SE* *<* .001, 95% CI (−0.06, −0.01)]. This indicates that those caregivers who reported lower initial goal achievement improved in their goal achievement at a faster rate compared to caregivers who initially reported higher initial goal achievement. The growth model of respite goal achievement is provided in Figure 1 demonstrating that individuals on average fell between neutral (3) and agree (4) at the beginning of the study (3.67), but increased by.032 on average each week, resulting in an estimated score between agree (4) and strongly agree (5) by the end of the study period (4.18).

### 3.2 Primary analysis

Multilevel models testing the primary hypothesis are presented in Table 3. We found that that there was a positive association between average goal achievement score and all three respite outcomes. This indicates that individuals who reported achieving their goals more consistently were more satisfied with their respite time-use (i.e., felt happier about their respite, and felt that they had received enough respite) and felt better because of their respite time-use (i.e., felt that their respite made them a better caregiver), relative to individuals with lower average weekly goal achievement scores. We also found significant positive within-person associations between weekly goal achievement and all respite weekly outcomes. This indicates that on weeks when individuals completed their respite goals, they reported feeling happier about their respite, feeling more like their respite made them a better caregiver, and feeling more like they had enough respite time, compared to weeks when they were less successful in their respite goal achievement. These associations were also present for the lagged within-person association between goal achievement and all respite time use outcomes. This indicates that better goal achievement for a given week, relative to a participant's typical goal achievement, predicts improvements in all the respite outcomes one week later.

**Table 3:** Multilevel model estimating experienced and evaluative time use outcomes.

Estimates	Happy with respite		Better caregiver because of respite		Enough respite	
	*b*	95% CI	*b*	95% CI	*b*	95% CI
Fixed effects						
Intercept	3.633		3.732		3.087	
Lagged dependent variable	−0.237**	−0.318, −0.155	−0.235***	−0.313, −0.156	−0.087	−0.184, 0.010
Average goal achievement	0.910***	0.749, 0.107	0.739***	0.556, 0.921	0.735***	0.490, 0.980
Weekly goal achievement	0.443***	0.314, 0.571	0.334***	0.232, 0.437	0.410***	0.296, 0.524
Lagged weekly goal achievement	0.167**	0.060, 0.276	0.096*	0.009, 0.183	0.158**	0.038, 0.277
Average respite time	0.002	−0.008, 0.009	0.001	−0.009, 0.011	0.012*	0.002, 0.024
Weekly respite time	0.011***	0.005, 0.017	0.008**	0.003, 0.013	0.021**	0.008, 0.034
Lagged weekly respite time	0.003	−0.001, 0.012	0.004	−0.003, 0.012	−0.002	−0.011, 0.006
Randomization group	0.064	−0.169, 0.298	0.031	−0.207, 0.271	0.196	−0.072, 0.464
Time	0.035**	0.013, 0.056	0.024**	0.007, 0.039	0.022***	0.001, 0.043
Variance parameters						
Random intercept	0.05	0.008, 0.287	0.117	0.063, 0.217	0.204	0.101, 0.412
Weekly goal achievement	0.09	0.043, 0.189	0.060	0.026, 0.140	0.034	0.010, 0.126
Lagged weekly goal achievement	0.02	0.001, 0.559	0.033	0.004, 0.273	0.052	0.016, 0.412
Autoregressive correlation	0.38	0.120, 0.589	0.331	0.097, 0.529	0.244	−0.170, 0.585
Residual variance	0.55	0.403, 0.761	0.380	0.295, 0.490	0.669	0.498, 0.899

Time measured in weeks; Respite time measured in hours.

* *p* < .05.

** *p* < .01.

*** *p* < .001.

All associations between goal achievement and respite time-use outcomes were significant above and beyond the effects of weekly respite time use. Average weekly respite time was positively associated with participants feeling like they got enough respite time, indicating that individuals who get more respite time during their weeks on average tended to feel that they get enough respite time compared to other individuals who report getting less respite time per week on average. However, average weekly respite time was not related to participants' average report of happiness with respite time and average report of feeling that respite makes them a better caregiver. There were also significant positive within person associations between weekly variation in respite time and the respite experience and evaluative outcomes. This indicates that when participants get more respite time on a given week relative to their typical amount of respite time, they report feeling happier with their respite, feeling more like their respite makes them a better caregiver, and feeling more like they got enough respite. However, this association was only significant for same-week associations between respite time and respite experience and evaluative outcomes. These associations were not significant for the lagged weekly respite time use, indicating that respite time use for one week is not predictive of respite experience and evaluation for the next week. Lastly, we also found a significant positive association between the time growth parameter and the experience and evaluative respite outcomes, indicating that participants experienced a consistent increase in feeling happy with their respite time, feeling like their respite made them a better caregiver, and feeling like they got enough respite every week across the course of using a respite time management app.

## 4 Discussion

The objective of the current study was to evaluate caregivers' experience and evaluation of their respite time-use. Consistent with our primary hypothesis, on weeks when caregivers reported improved goal achievement, meaning they did the type of activity that they wanted to do or had planned to do, they reported improvements in their evaluation and experience of their respite during the same week, as well as exhibited sustained improvements one week later. While total amount of respite received in a week was also associated with better evaluation and experience of respite for the same week, it was not predictive of the experience and evaluation of their respite the next week. Simply put, when caregivers achieved their respite goals, they reported that they were happier with their respite and that their respite helped them be a better caregiver independent of the amount of respite time they were able to engage in. These results suggest that by consistently setting, completing, and reviewing goals about how to spend their respite time benefits caregivers' ability to achieve their goals, while strengthening their subjective experience of respite (34, 35, 44). The TLC app, which uses repeated goal-setting and goal-review techniques to help caregivers identify and then do the kinds of activities they *want* to do during their respite (no matter how limited or abundant), maybe an effective intervention, by maximizing the benefit of respite for caregivers.

### 4.1 Clinical implications

As caregivers are at heightened risk for physical and mental health issues (3, 8), there is considerable need to provide and improve strategies to reduce the elevated stress and burden that caregivers experience. Technology-delivered interventions – i.e., those delivered through automated telephone prompts, digital applications (“apps”), and/or interactive websites – are becoming increasingly common, representing a possible cost-efficient and effective way to provide support, education, and information to family caregivers (45, 46). Consistent with recommendations from a National Institutes of Health research summit (32), widely available mobile and internet technologies hold potential for older users who are increasingly able and willing to use computer-based or internet-delivered supports (47). Within the larger TLC research study and within these current time-use analyses, we found that when given a structure to review and reflect on respite time-use, participants improved throughout the course of the study in their ability to achieve their respite goals, supporting that computer-based programs such as the TLC app can aid in the development of adaptive behavioral change that is beneficial to the daily lives of caregivers.

Over half of family caregivers say online tools have been helpful to their ability to cope with the stress of being a caregiver (48). While online-delivered interventions or “apps” may not be suitable or preferable for all caregivers, especially for those without access to high-speed internet or computer technology and those who are not interested in engaging with these types of technologies (49), such limitations are likely outweighed by the strengths, particularly their ability to deliver support to hard-to-reach populations, such as caregivers who may not be available to engage in traditional educational or supportive services (46); who cannot access services during normal business hours; or who live in rural and remote areas where such services may not be available. Use of technology-delivered interventions have been shown to decrease loneliness, increase perceived social support, and address feelings of burden among family caregivers (51). This study's findings add to the potential benefits of technology-delivered interventions and should increasingly be considered as one tool for supporting family caregivers in improving their respite time-use to maximize the benefits that respite provides.

### 4.2 Limitations

The current study has important limitations to consider. First, the sample comprised a higher proportion of white caregivers with adequate incomes compared to national surveys of caregiver demographics and socio-economic characteristics. Therefore, results may not generalize to populations with more diverse backgrounds or those with fewer socio-economic resources. In particular, digital inequality and computer literacy may be important factors to consider regarding use and feasibility of an online intervention for this population [See (47)]. Future research should strive to include more diverse samples in evaluating the use of this type of technology with older populations of caregivers to ensure generalizability of results, and to advance the science of user design principles for an older population (i.e., multimedia instructions, accessible user-interfaces). Second, the current study did not include a control group in relation to goal setting and achievement as both groups in the study were provided with access to the TLC mobile app, albeit on different schedules. Because of the lack of manipulation to respite goal achievement process, such as comparing individuals to groups who were not instructed to engage with goal-oriented processes for respite time, associations reported here may be influenced by factors that were not assessed in this study.

### 4.3 Future directions

Results from the current study provide support that when caregivers are more successful in achieving their respite goals, their subjective evaluation of and experience with respite improves. Several avenues of research would benefit current efforts to improve mental health resources of caregivers when considering these findings. There remains a need to evaluate public education and dissemination methods for web-based applications to improve awareness and motivation for engaging with app-based interventions. Additionally, more research is needed on identifying who may benefit most from respite goal setting and -reviewing activities. Identifying characteristics of caregivers and their circumstances, such as demographics, severity of care recipient condition, and the type of caregiver burden they experience, would allow for more precise targeting of beneficiaries, as well as identification of barriers that prevent individuals to effectively use respite. Overall, web-based smartphone applications show promise for aiding the structuring and experience of caregivers' much needed respite time via goal-setting mechanisms.

## Data Availability

The datasets presented in this study can be found in online repositories. The names of the repository/repositories and accession number(s) can be found below: Hive Repository, University of Utah. https://doi.org/10.7278/S50d-2rgg-4549.
